# Configuration-Dependent Optimal Impedance Control of an Upper Extremity Stroke Rehabilitation Manipulandum

**DOI:** 10.3389/frobt.2018.00124

**Published:** 2018-11-01

**Authors:** Borna Ghannadi, Reza Sharif Razavian, John McPhee

**Affiliations:** Systems Design Engineering, University of Waterloo, Waterloo, ON, Canada

**Keywords:** optimal impedance control, linear quadratic regulator, operational space, rehabilitation manipulandum, human-robot interaction, stroke rehabilitation

## Abstract

Robots are becoming a popular means of rehabilitation since they can decrease the laborious work of a therapist, and associated costs, and provide *well-controlled* repeatable tasks. Many researchers have postulated that human motor control can be mathematically represented using optimal control theories, whereby some cost function is effectively maximized or minimized. However, such abilities are compromised in stroke patients. In this study, to promote rehabilitation of the stroke patient, a rehabilitation robot has been developed using optimal control theory. Despite numerous studies of control strategies for rehabilitation, there is a limited number of rehabilitation robots using optimal control theory. The main idea of this work is to show that impedance control gains cannot be kept constant for optimal performance of the robot using a feedback linearization approach. Hence, a general method for the real-time and optimal impedance control of an end-effector-based rehabilitation robot is proposed. The controller is developed for a 2 degree-of-freedom upper extremity stroke rehabilitation robot, and compared to a feedback linearization approach that uses the standard optimal impedance derived from covariance propagation equations. The new method will assign optimal impedance gains at each configuration of the robot while performing a rehabilitation task. The proposed controller is a linear quadratic regulator mapped from the operational space to the joint space. Parameters of the two controllers have been tuned using a unified biomechatronic model of the human and robot. The performances of the controllers were compared while operating the robot under four conditions of human movements (impaired, healthy, delayed, and time-advanced) along a reference trajectory, both in simulations and experiments. Despite the idealized and approximate nature of the human-robot model, the proposed controller worked well in experiments. Simulation and experimental results with the two controllers showed that, compared to the standard optimal controller, the rehabilitation system with the proposed optimal controller is assisting more in the active-assist therapy while resisting in active-constrained case. Furthermore, in passive therapy, the proposed optimal controller maintains the position error and interaction forces in safer regions. This is the result of updating the impedance in the operational space using a linear time-variant impedance model.

## 1. Introduction

### 1.1. Motivation

Upper extremity motor impairments are common among post-stroke patients. If the rehabilitation therapy is stimulating and intense, it will be effective in treatment of disabilities (Richards and Malouin, 2015). Thus, upper extremity rehabilitation robots including robotic manipulanda^1^ (“InMotion Arm” and “ReoGo”) and robotic exoskeletons (“ArmeoPower,” and “ArmeoSpring”) have been commercially introduced to the clinical setting (Maciejasz et al., 2014; Proietti et al., 2016). Although, in some studies, advantages of these robots over traditional therapy methods are minor (Brewer et al., 2007; Wisneski and Johnson, 2007; Lo et al., 2010; Mazzoleni et al., 2013; Maciejasz et al., 2014), their use cannot be ignored since they can provide well-controlled repeatable tasks, progress evaluation measurements and entertaining user-interfaces (Reinkensmeyer, 2009; Kowalczewski and Prochazka, 2011).

When stroke management is supported by effective care, rehabilitation costs can be substantially reduced (Krueger et al., 2012). Effective stroke care includes rapid assessment and rehabilitation with efficient outcomes in physical and functional recovery (Hebert et al., 2016). Efficient physical recovery is a qualitative measure, and a healthy subject is assumed to have an efficient physical activity level. Hence, if a stroke rehabilitation approach can improve the physical activity of a stroke patient to the level of a healthy subject, the rehabilitation can be considered effective, i.e., it cannot do any better.

Studies have shown that a human interacts with the environment while minimizing an error and effort or, in general, a cost function (Todorov and Jordan, 2002; Franklin et al., 2008). In other words, the human's central nervous system (CNS) optimally controls human interaction. In rehabilitation therapy, there is an interaction between the stroke patient and a therapist or robot (or in general, an environment). To promote effective therapy, if the stroke patient's CNS cannot maintain the optimality goal, this internal optimal control problem should be solved externally with the aid of assistive devices (Jarrassé et al., 2012). Thus, we assume that the use of optimal control methods in rehabilitation robotics is well-suited to assisting an impaired CNS. This assumption is consistent with previous studies, such as Hunt et al. (1999) who used optimal control theory in a feedback balance control mechanism to maintain standing of paraplegic subjects, Emken et al. (2005) who considered rehabilitation robot training as an optimization problem and designed an optimal controller for assist-as-needed (active-assisted) therapy, Ibarra et al. (2014) and Ibarra et al. (2015) who developed an optimal controller for ankle rehabilitation, Mombaur (2016) who uses optimal control theory to predict natural (healthy human) movement and improve the device performance in rehabilitation technologies, Wang et al. (2017) who used optimal control to maintain patient's safety and comfort during elbow rehabilitation, and Corra et al. (2017) who implemented optimal control to adjust the gains of a controller for arm rehabilitation.

### 1.2. Control strategies in rehabilitation robotics

Control strategies for rehabilitation robots can be divided into two general subgroups: (1) High-level control scenarios for stimulating neural plasticity, and (2) Low-level control scenarios to implement high-level scenarios (Maciejasz et al., 2014). High-level control scenarios include: assistive, corrective (coaching) and resistive (challenge-based) control modes. Among these modes, assistive control is the core element in post-stroke rehabilitation therapy. In assistive mode, three types of low-level control scenarios are implemented on these robots: (1) Passive control, (2) Triggered passive control, (3) Partially assistive control. Passive trajectory tracking and impedance-based control methods, which are types of passive and partially assistive control scenarios, respectively, are widely used in these robots (Maciejasz et al., 2014; Proietti et al., 2016).

In robotic rehabilitation, because of physical interaction of the patient with a mechanical device, safety is a fundamental element in the design of a low-level control scenario. Thus, impedance-based control scenarios are more applicable for robotic rehabilitation (Marchal-Crespo and Reinkensmeyer, 2009; Maciejasz et al., 2014; Proietti et al., 2016), since conventional position/force control scenarios (passive trajectory tracking) do not consider dynamic interaction of the human-robot system (Hogan, 1985). Furthermore, assist-as-needed therapy, which encourages voluntary participation of the patient, is implementable through the impedance-based control scenarios.

In impedance-based control, the amount of assistance/resistance (i.e., compliance) can be adjusted by controlling the impedance gains. However, in the presence of a variable admittance environment (i.e., different patients) or different trajectories (i.e., robot configurations), the interaction force and configuration will exacerbate inefficiency of the controller with non-optimal gains. For example, a resistive-capacitive impedance control with therapist-adjustable constant stiffness and damping ratios is implemented in the upper extremity rehabilitation manipulandum from the Toronto Rehabilitation Institute (TRI) and Quanser Consulting Inc., but these gains cannot be adjusted *optimally* using trial and error by the therapist (Huq et al., 2012).

Besides other methods of partially assistive control (e.g., attractive force-field control, model-based assistance, learning-based assistance, counter-balance-based assistance, and performance-based adaptive control), adaptive and optimal forms of impedance control have been developed to deal with variable admittance environments. Hussain et al. (2013) used an adaptive impedance control for patient-cooperative therapy of a lower-limb exoskeleton, and they verified the controller performance using an experimental setup. In more recent studies, optimal impedance controls for an exoskeleton gait trainer and elbow rehabilitation robot were developed (Dos Santos and Siqueira, 2016; Wang et al., 2017). The proposed methods were implemented in a computer simulation, and the real-time performance of the controllers was not discussed. In an exoskeleton, the impedance control is defined in the joint space, while in a manipulandum, the impedance model is in the operational space. Thus, the controllers developed for exoskeletons are not suitable for a manipulandum. Furthermore, exoskeleton controllers are developed for some sort of predefined rhythmic motions and they are not implementable for random reaching movements. Beside recent studies on exoskeletons, Maldonado et al. (2015) used stiffness-based tuning for an adaptive impedance control of an upper extremity manipulandum; the method was verified using computer simulations only and its real-time capabilities were not mentioned.

In some studies, to improve impedance control performance, the compliance has been controlled by an outer-loop force control (Erol and Sarkar, 2007; Siciliano et al., 2009; Ghannadi et al., 2014a). Depending on the controller structure and use of series elastic actuators, the compliance term can be controlled by an inner-loop force control in the presence of an outer-loop impedance control. For example, Perez-Ibarra et al. (2017) used an H-infinity force control to implement this approach. This hybrid impedance-force controller can be implemented by different methods such as weighted sum (Moughamir et al., 2005) or robust Markovian approach (Jutinico et al., 2017). However, this method only controls the compliance (i.e., interaction force) term, and the impedance gains are not optimal.

In a recent study, to select optimal target impedance for a lower limb exoskeleton, a method for estimating human admittance using particle swarm optimization was proposed (Taherifar et al., 2017). Overall, a general solution for an optimal impedance problem can be obtained with optimization techniques (i.e., an optimal control approach). Such techniques can adapt to variable admittance environments and different robot configurations. However, real-time control of the system limits the utilizable non-linear optimization methods. Ding et al. (2010) used a musculoskeletal human model (without including muscle dynamics) together with surface elecromyography (sEMG) signals to implement model-based assistance control on a rehabilitation *exoskeleton*. In Ghannadi et al. (2017a), we used a nonlinear model-predictive approach to control human-robot interaction in an upper extremity manipulandum. The method was verified using computer simulation, but experimental tests were not performed because of inefficient computation for real-time implementation.

Learning-based methods can also be used to evaluate the optimal impedance gains (Ge et al., 2014; Modares et al., 2016). Ge et al. (2014) implemented an adaptive linear quadratic regulator (LQR) to estimate the impedance gains, and Modares et al. (2016) used reinforcement learning to solve an LQR problem and achieve optimal impedance gains. However, the validity of the proposed methods was verified using simulation studies, and real-time implementation was not discussed. Other than LQR, H-infinity control approaches can be used to achieve optimal performance. In Kim et al. (2015), an H-infinity impedance control is implemented for an upper extremity exoskeleton. Compared to an LQR controller, the H-infinity controller is more robust because it can handle uncertainties in the impedance model. Design of an H-infinity controller depends on the selection of a weighting function, whereas in an LQR control, the optimal state feedback gain matrix is favorable. Thus, initial design of an H-infinity approach may take more effort than an LQR controller. Furthermore, H-infinity may have large numerical variations that require increased numerical precision, thereby increasing the computation cost for real-time implementation (Glover and Packard, 2017).

Since multi-link manipulanda are controlled in the joint space to achieve the desired impedance at the end-effector in the operational space, the optimal impedance gains should be assigned to the different robot configurations. For different configurations, the manipulability ellipsoid in robotics is introduced to determine the easiest manipulation direction (Yamashita, 2014). Thus, a method is required to optimally change the impedance gains based on the robot's manipulability ellipsoid. Hogan (2017) proposed an optimal impedance control for a one-dimensional system, the standard optimal impedance control (SOIC), which minimizes an cost function with position and force penalty. This problem was solved using covariance propagation equations. To the knowledge of the authors, there is no other optimal impedance control approach that has resolved different robot configuration problem independently.

### 1.3. Research objective

The control input for a conventional impedance control using nonlinear feedback linearization is in terms of the interaction force, which is defined based on the impedance model with time-invariant gains (see Appendix C). There is a trade-off between tracking accuracy and interaction force in the operational space, and increasing one of them may decrease the other. An impedance model (i.e., gains) can regulate this trade-off efficiently if they are adjusted optimally for different robot configurations. Tuning this trade-off is important since this can help the patient to safely (i.e., with an optimally safe-zoned interaction force) follow a desired trajectory with optimal accuracy. As discussed in section 1.2, different studies have tried to provide the best trade-off in robotic rehabilitation. However, there is a lack of research in the design of *real-time* optimal impedance control for different configurations of rehabilitation manipulanda. Furthermore, previous low-level controls of rehabilitation manipulanda have not included human-robot interactions for the adjustment of the robot controller.

To find optimal impedance gains, we restate the problem definition using optimal control theory: the best trade-off between tracking error and interaction force in the operational space can be revisited as finding an optimal control law that minimizes the tracking and effort error in the operational space. If this control law can satisfy the impedance model with time-variant gains, optimal target impedance will be achieved because these gains are the resulting optimal solution. The objective of this work is to design a general real-time optimal impedance control for rehabilitation manipulanda. This controller is designed to reduce therapist intervention (with fewer gain adjustments) and improve the quality of therapy in terms of safety (less interaction force based on robot manipulability) and rehabilitation (optimal tracking).

In our previous study, we presented an optimal impedance control (OIC) for an upper extremity stroke rehabilitation robot (Ghannadi and McPhee, 2015); adjustment and performance-evaluation of the controller were done by simulating the robot interacting with a musculoskeletal upper extremity model (Ghannadi et al., 2014b). The current paper is an extension to our previous study. Here, a general method that optimally adjusts impedance gains for variable robot configurations is developed and tuned by simulating the human-robot system. The proof that justifies the existence of a linear time variant (LTV) impedance model is provided. The controller is implemented on a Quanser Consulting Inc./TRI robot. Then, the performance of the controller in terms of interaction force and tracking accuracy is evaluated and compared to the SOIC (Hogan, 2017) through simulations and experiments. In experiments, a complete dynamic model of the robot including joint and end-effector frictions, and joint stiffness are considered.

This paper is organized as follows. First, in the section 2, the modeling procedure and controller design are presented. Second, in the section 3, simulation and experiment descriptions and assessment criteria are provided. Next, in the section 4, OIC simulation and experimental results are compared to SOIC. Finally, in the section 5, contributions and future work are presented.

## 2. Modeling and control

In this section, first, the human-robot system model (which is used in a model-in-loop simulation) is described. Next, the proposed controller design is discussed.

### 2.1. Model development

**Figure 1:**
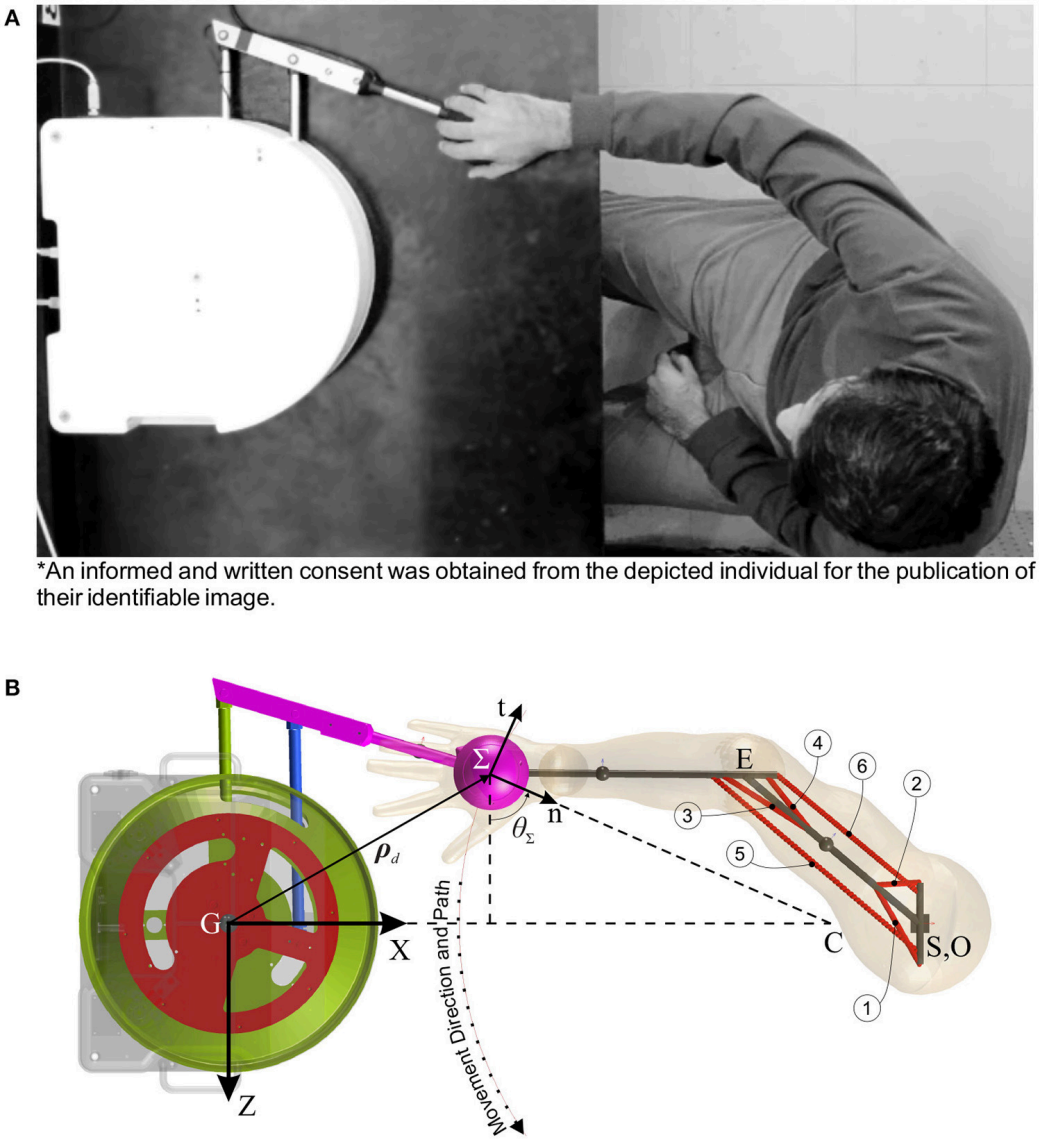
Human-robot rehabilitation system. **(A)** Experimental setup (an informed and written consent was obtained from the depicted individual for the publication of their identifiable image). **(B)** MapleSim™model (circled numbers show the corresponding muscle number).

The upper extremity stroke rehabilitation robot is a 2 degree-of-freedom (DOF) parallelogram arm that moves the hand in the horizontal plane to perform reaching movements for therapy (Figure 1B). This robot is driven by two DC motors that share the same axis of rotation, and are connected to the 2 DOF arm through disc and timing belt mechanisms. Since the proposed controller is particularly suited for backdrivable robots, the simulation model of the rehabilitation robot is assumed to have negligible frictional forces so that the robot can be backdriven. Hence, it is modeled as a frictionless planar parallelogram linkage in the MapleSim™ software package.

The musculoskeletal arm is considered a planar 2 DOF linkage with 19 muscles lumped in 6 muscle groups (Ghannadi et al., 2014b) (Figure 1A). In this model, upper extremity tendons were treated as rigid elements^2^, and the passive elements of the arm muscles were assumed to have less contribution than the active elements in muscle forces. Hence, the contractile element of the Hill-type muscle model is used to model muscle dynamics, and forward static optimization (Ghannadi et al., 2014b) is implemented to solve the muscle force sharing problem while tracking the desired curvilinear path (Zadravec and Matjačić, 2013) (Figure 1A) with minimum jerk and a bell-shaped tangential speed (Flash and Hogan, 1985) under robot operation. This musculoskeletal arm is also developed in the MapleSim™ software package.

These two models are integrated in MapleSim™ and connected to each other by a free rotational revolute joint with a force sensor. There are eight inputs to the human-robot system consisting of two robot motor torque inputs (*T*_*R*_1,2__) and six muscle activations (*a*_1..6_). In this system, the number of outputs is six, where two are from motor encoders (*q*_1,2_), two are from the force sensor (*F*_*ext*_*Z, X*__), and two are the musculoskeletal model joint angles (θ_1,2_).

In contrast to admittance control, impedance control can be used for backdrivable systems. Thus, for implementing the proposed optimal controller, we assume that the friction is negligible so that the robot can be backdriven. In simulations, the robot model has no friction and the musculoskeletal model has only approximate parameters for the muscles and inertial properties; thus, we do not expect a close quantitative match between simulation and experimental results. Nevertheless, the model will be effective for the design and tuning of a feedback controller if a good qualitative match between simulation and experimental results is achieved.

### 2.2. Optimal control method

In an optimal control structure, it is desired to carry out a desired task while minimizing a cost function. The dynamic equation of the robot excluding frictional forces is as follows:

(1) $$\begin{array}{c} T_{R}-J_{R}^{T}F_{int}=M_{R}(q)\ddot{q}+C_{R}(q,\dot{q})\dot{q}=\;\Gamma_{R}(q,\dot{q},\ddot{q}) \end{array}$$

where **T**_*R*_ is the vector of robot motor torques, and **J**_*R*_ is the robot geometric Jacobian. **F**_*int*_ is the robot to human interaction force in the global coordinates, and it is equal to the measured force by the force sensor (i.e., **F**_*int*_ = **F**_*ext*_). **M**_*R*_ is the robot inertia (mass) matrix, and **C**_*R*_ is the robot Coriolis-centrifugal matrix. The state-space representation for the robot dynamics can be expressed as:

(2) $$\begin{array}{c} {\dot{x}}_{q}=\left\{\begin{array}{c} {\dot{q}}_{1}\\ {\dot{q}}_{2}\\ M_{R}^{-1}(u-\Gamma_{R}(q,\dot{q},0)) \end{array}\right\}=F(x_{q},u)\text{,} \end{array}$$

where:

(3) $$\begin{array}{c} u=T_{R}-J_{R}^{T}F_{ext}\text{,} \end{array}$$

and

(4) $$\begin{array}{c} x_{q}=\left\{\begin{array}{c} q\\ \dot{q} \end{array}\right\}=\left\{\begin{array}{c} q_{1}\\ q_{2}\\ {\dot{q}}_{1}\\ {\dot{q}}_{2} \end{array}\right\}\text{.} \end{array}$$

The objective is to develop a real-time controller that optimizes impedance gains at different configurations. Since the state-space representation (2) is nonlinear, application of nonlinear optimal control approaches will be limited by the computation time. On the other hand, if (2) was linear, a linear optimal controller (such as LQR or H-infinity) could solve this problem in real-time. Since the robot performs preplanned point to point reaching tasks in the horizontal plane (Lu et al., 2011), we can perform Jacobian linearization on the robot dynamics along the preplanned rehabilitation trajectory to apply a systematic linear control technique, which can allow for real-time control. In recent robotic rehabilitation simulation studies (Ge et al., 2014; Modares et al., 2016), LQR was used to implement optimal impedance control. Hence, we also use an LQR approach to solve the optimal impedance control problem. The LTV state-space equation of the robot's error dynamics will be:

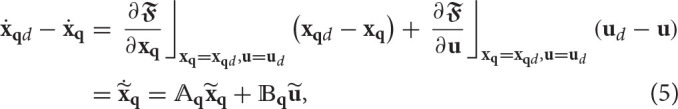



where subscript *d* indicates the desired value of a variable, and accent ~ denotes the error of the desired variable with respect to its actual value. A and B are the state and input matrices, respectively. The desired control input is defined by the following equation:

(6) $$\begin{array}{c} u_{d}=\Gamma_{R}\left(q_{d},{\dot{q}}_{d},{\ddot{q}}_{d}\right). \end{array}$$

At each operational point, which is defined every 1000/ν ms of the rehabilitation trajectory, the model is linearized and the interaction force is applied to the robot. ν is the sampling-time frequency which is measured in Hz. It is worth noting that, if very few operational points are defined, the system may be biased into optimizing for static situations. At each operational point, the controllability (C) and observability (O) matrices are defined as:

(7) $$\begin{array}{l} C={\left[\begin{array}{c} {\text{B}}_{q}{\text{ B}}_{q}{\text{B}}_{q}{\text{ A}}_{q}^{2}{\text{B}}_{q}{\text{ A}}_{q}^{3}{\text{B}}_{q} \end{array}\right]}_{4\times 8}\text{, }O={\left[\begin{array}{c} \begin{array}{l} \text{I}\\ {\text{A}}_{q}\\ {\text{A}}_{q}^{2}\\ {\text{A}}_{q}^{3} \end{array} \end{array}\right]}_{16\times 4}\text{,} \end{array}$$

where I is an identity matrix.

At each operational point, there is an LTV impedance model which is relating the end-effector operational space error (${\tilde{\rho }}^{\Sigma }$) to interaction force error:

(8) $$\begin{array}{c} -{\tilde{F}}_{ext}^{\Sigma }=M_{imp}{\tilde{\ddot{\rho }}}^{\Sigma }+B_{imp}{\tilde{\dot{\rho }}}^{\Sigma }+K_{imp}{\tilde{\rho }}^{\Sigma }\text{,} \end{array}$$

here, it is assumed that the desired interaction force is equal to zero, that is ${\tilde{\text{F}}}_{ext}^{\Sigma }=-{\text{F}}_{ext}^{\Sigma }$. $\tilde{\rho }$ is the end-effector position error in the Cartesian coordinates, and subscript *imp* stands for the impedance model. **M**, **B**, and **K** are mass, damping, and stiffness coefficients (impedance gains), respectively. These gains are time-dependent. These gains are time-dependent. Superscript Σ denotes that the corresponding vector is defined in the end-effector's n-t coordinates (i.e., the operational space; see Figure 1B). If **R**_Σ_ is the rotation matrix transforming the n-t coordinates to the Cartesian coordinates, ${\tilde{\rho }}^{\Sigma }$ can be obtained from the following equations:

(9) $$\begin{array}{c} \{\begin{array}{l} {\tilde{\rho }}^{\Sigma }=R_{\Sigma }^{T}\tilde{\rho }\text{,}\\ R_{\Sigma }=\left[\begin{array}{cc} \cos(\theta_{\Sigma }) & -\sin(\theta_{\Sigma })\\ \sin(\theta_{\Sigma }) & \cos(\theta_{\Sigma }) \end{array}\right]\text{,} \end{array} \end{array}$$

where θ_Σ_ is defined in Figure 1B.

The LTV state-space Equation (5) is in terms of errors, so we can use the infinite time^3^ LQR to optimally control the robot along the desired trajectory. For the LQR approach, the quadratic cost function is:

(10) $$\begin{array}{c} J_{q}=\frac{1}{2}\int_{0}^{\infty }\left({\tilde{x}}_{q}^{T}{\text{Q}}_{q}{\tilde{x}}_{q}+\;{\tilde{u}}^{T}{\text{R}}_{q}\tilde{u}\right)dt\text{.} \end{array}$$

The above cost function is for minimizing the joint space error together with the consumed energy. An impedance control approach controls the robot performance in the operationals space as in (8) (Siciliano et al., 2009; Hogan, 2017). Thus, for an optimal impedance control it will be desired to minimize the operational space error together with the operationally applied force (effort) error while satisfying (8). In other words, the following cost function is more appropriate than (10):

(11) $$\begin{array}{c} J_{\Sigma }=\frac{1}{2}\int_{0}^{\infty }\left({\tilde{x}}_{\Sigma }^{T}{\text{Q}}_{\Sigma }{\tilde{x}}_{\Sigma }+\;{\tilde{\text{F}}}_{\Sigma }^{T}{\text{R}}_{\Sigma }{\tilde{\text{F}}}_{\Sigma }\right)dt\text{,} \end{array}$$

where ${\tilde{F}}_{\Sigma }$ is the operational space transformation of the applied force error in the Cartesian space ($\tilde{F}$):

(12) $$\begin{array}{c} \{\begin{array}{l} \begin{array}{c} \text{F}=J_{R}^{-T}u=J_{R}^{-T}T_{R}-F_{ext}\text{,}\\ \tilde{\text{F}}≊J_{Rd}^{-T}\;\tilde{u}\text{.} \end{array}\; \end{array} \end{array}$$

To solve the LQR problem with the updated cost function in (11), we use the mapping from the operational space into the joint space and then solve the LQR problem with the ordinary cost function in (10).

#### 2.2.1. Mapping the operational into joint space

We define the joint, Cartesian and operational state errors as follows:

(13) $$\begin{array}{c} {\tilde{x}}_{q}=\left\{\begin{array}{c} q_{d}-q\\ {\dot{q}}_{d}-\dot{q} \end{array}\right\}=\left\{\begin{array}{c} \tilde{q}\\ \tilde{\dot{q}} \end{array}\right\}\text{,} \end{array}$$

(14) $$\begin{array}{c} {\tilde{x}}_{\rho }=\left\{\begin{array}{c} \rho_{d}-\rho \\ {\dot{\rho }}_{d}-\dot{\rho } \end{array}\right\}=\left\{\begin{array}{c} \tilde{\rho }\\ \tilde{\dot{\rho }} \end{array}\right\}\text{,} \end{array}$$

(15) $$\begin{array}{c} {\tilde{x}}_{\Sigma }=\left\{\begin{array}{c} \rho_{d}^{\Sigma }-\rho^{\Sigma }\\ {\dot{\rho }}_{d}^{\Sigma }-{\dot{\rho }}^{\Sigma } \end{array}\right\}=\left\{\begin{array}{c} {\tilde{\rho }}^{\Sigma }\\ {\tilde{\dot{\rho }}}^{\Sigma } \end{array}\right\}\text{.} \end{array}$$

Based on the inverse kinematics of the robot, the geometric Jacobian definition (Siciliano et al., 2009) and first-order Taylor series expansion, the relation between the Cartesian and joint space errors can be defined as:

(16) $$\begin{array}{c} \{\begin{array}{l} \tilde{\rho }≊J_{Rd}\tilde{q}\text{,}\\ \tilde{\dot{\rho }}≊{\dot{J}}_{Rd}\tilde{q}+J_{Rd}\tilde{\dot{q}}\text{,} \end{array} \end{array}$$

Thus, the operational state error in terms of the joint state error can be defined by the following equation:

(17) $$\begin{array}{c} {\tilde{x}}_{\rho }≊\left[\begin{array}{cc} J_{Rd} & 0\\ {\dot{J}}_{Rd} & J_{Rd} \end{array}\right]{\tilde{x}}_{q}\text{.} \end{array}$$

Consider Figure 1B, the operational coordinate (Σ:n-t) is the rotated and translated Cartesian coordinate (G:ZX) by angle θ_Σ_ and desired position vector *ρ*_*d*_, respectively; thus, the relation between the operational and Cartesian space errors can be defined as:

(18) $$\begin{array}{c} \{\begin{array}{l} \tilde{\rho }=R_{\Sigma }{\tilde{\rho }}^{\Sigma }\text{,}\\ \tilde{\dot{\rho }}={\dot{R}}_{\Sigma }{\tilde{\rho }}^{\Sigma }+R_{\Sigma }{\tilde{\dot{\rho }}}^{\Sigma }\text{.} \end{array} \end{array}$$

By defining *ϖ*_Σ_ as the skew symmetric matrix of the angular velocity (${\overset{\cdot }{\theta }}_{\Sigma }$), the operational state error can be defined in terms of the Cartesian state error as:

(19) $$\begin{array}{c} {\tilde{x}}_{\rho }=\left[\begin{array}{cc} R_{\Sigma } & 0\\ \varpi_{\Sigma }R_{\Sigma } & R_{\Sigma } \end{array}\right]{\tilde{x}}_{\Sigma }\text{.} \end{array}$$

Finally, the operational and joint state errors can be related as:

(20) $$\begin{array}{c} {\tilde{x}}_{\Sigma }≊{\left[\begin{array}{cc} R_{\Sigma } & 0\\ \varpi_{\Sigma }R_{\Sigma } & R_{\Sigma } \end{array}\right]}^{-1}\left[\begin{array}{cc} J_{Rd} & 0\\ {\;}_{Rd} & J_{Rd} \end{array}\right]\;{\tilde{x}}_{q}=\left[\begin{array}{cc} J_{Rd}^{\Sigma } & 0\;\\ {\dot{J}}_{Rd}^{\Sigma } & J_{Rd}^{\Sigma } \end{array}\right]\;{\tilde{x}}_{q}=\;T_{q}^{\Sigma }\;{\tilde{x}}_{q}\text{.} \end{array}$$

#### 2.2.2. Building updated LQR matrices

We define Q_Σ_ and R_Σ_ in the operational space cost function (11) as positive definite diagonal matrices. Using the mapping Equation (20) we can correlate the first terms of the two quadratic cost functions (10,11); thus, Q_**q**_ can be defined as:

(21) $$\begin{array}{c} {\text{Q}}_{q}\;=\;{\left(T_{q}^{\Sigma }\right)}^{T}\;{\text{Q}}_{\Sigma }T_{q}^{\Sigma }\text{.} \end{array}$$

Note that Q_**q**_ is positive definite, since Q_Σ_ is positive definite. Since:

(22) $$\begin{array}{c} {\tilde{\text{F}}}_{\Sigma }=R_{\Sigma }\tilde{\text{F}}\text{,} \end{array}$$

considering (12) we can also rearrange the energy term in the joint space cost function (10) as:

(23) $$\begin{array}{l} {\tilde{u}}^{T}{\text{R}}_{q}\tilde{u}={\left(J_{Rd}^{T}\tilde{\text{F}}\right)}^{T}{\text{R}}_{q}\left(J_{Rd}^{T}\tilde{\text{F}}\right)={\left(J_{Rd}^{T}R_{\Sigma }\;{\tilde{\text{F}}}_{\Sigma }\right)}^{T}{\text{R}}_{q}\left(J_{Rd}^{T}R_{\Sigma }\;{\tilde{\text{F}}}_{\Sigma }\right)\;\\ \;={\tilde{\text{F}}}_{\Sigma }^{T}R_{\Sigma }^{T}J_{Rd}{\text{R}}_{q}J_{Rd}^{T}R_{\Sigma }{\tilde{\text{F}}}_{\Sigma \;}⩾\;0. \end{array}$$

Now since R_Σ_ is positive definite, if:

(24) $$\begin{array}{c} {\text{R}}_{q}={\left(R_{\Sigma }^{T}J_{Rd}\right)}^{-1}{\text{R}}_{\Sigma }{\left(J_{Rd}^{T}R_{\Sigma }\right)}^{-1}={\left(J_{Rd}^{\Sigma }\right)}^{-1}{\text{R}}_{\Sigma }{\left(J_{Rd}^{\Sigma }\right)}^{-T}\text{.} \end{array}$$

R_**q**_ is also positive definite unless the robot is at a singularity point. Based on (24), minimizing the energy term in the joint space cost function (10) will indirectly minimize the energy term in the operational space cost function (11).

#### 2.2.3. Optimal impedance control

**Figure 2:**
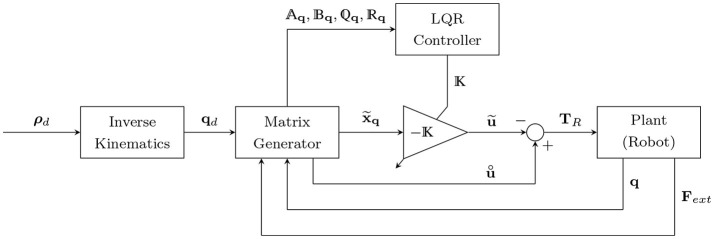
Optimal impedance controller scheme. *ρ*_*d*_ and **q**_*d*_ are the desired positions in the operational and joint spaces, respectively. ${\tilde{\text{x}}}_{\text{q}}$ is the state error vector in the joint space, $\tilde{\text{u}}$ is the optimal control input for the error dynamics, and $\ddot{\text{u}}$ is the nominal control input (desired torque minus the torque caused by the interaction force). *A*_**q**_, *B*_**q**_, and Q_**q**_ and R_**q**_ are the time-varying state, input, and LQR gain matrices, respectively.

With the updated LQR matrices, the optimal impedance controller scheme takes the structure shown in Figure 2. Using (3), the driving torque will be:

(25) $$\begin{array}{c} T_{R}=u+J_{R}^{T}F_{ext}\text{,} \end{array}$$

where the control input **u** is defined such that it should optimally control the error dynamics (by $-\tilde{\text{u}}$) while applying the nominal control input ($\overset{{}^{\circ}}{\text{u}}$). Thus, it will have the following form:

(26) $$\begin{array}{c} \left\{ \begin{array}{l} u\overset{\Delta }{=}\overset{\circ }{u}-\tilde{u}\text{,}\\ \overset{\circ }{u}=u_{d}-J_{Rd}^{T}F_{ext}\text{,}\\ \tilde{u}=-\text{K}{\tilde{x}}_{q}\text{.} \end{array}\right. \end{array}$$

Note that the nominal control input is equal to the desired system dynamics (desired control input) minus the torque caused by the interaction force at any desired location. This subtraction (${\text{u}}_{d}-{\text{J}}_{Rd}^{T}{\text{F}}_{ext}$) at a zero tracking error will lead to a zero desired interaction force in (8). Finally, the Equations (25), (26) are used to satisfy the impedance model (8) (as shown in Appendix A), in order to overcome the robot dynamics and interaction force.

## 3. Controller assessment

Here, the simulation procedure for controller tuning, assessment and comparison is presented. Then, the experimental procedure for validation of the simulation results is discussed. Finally, assessment criteria for controller evaluation are provided.

### 3.1. Simulations

In robotic rehabilitation, it is usually desired to follow a predefined path. During a path-following task, at least three therapy cases can occur (Ding et al., 2007; Amirabdollahian, 2011):

1. *Passive* case: the patient cannot accomplish the task, so the robot actively manipulates the patient's hand.
2. *Active-assisted* case: the patient is unable to finish the task independently in a specified time interval. Thus, the robot assists the patient as needed.
3. *Active-constrained* case: the patient can accomplish the task independently even faster than the predefined time interval. Hence, the robot tries to resist against patient's rapid movements.

Here, to evaluate the performance of the controller during a rehabilitation procedure, four modes of movement are considered: impaired, healthy, delayed and time-advanced hand movement along the specified path. Each of these modes will lead to one of the above therapy cases.

In the impaired hand movement mode, the upper extremity of the patient is totally dysfunctional (zero muscle activation), so the passive case will occur. In the healthy mode, the patient has normal timing and coordination, so one of the active cases can happen depending on the healthy subject performance. The delayed hand movement mode is used to model a stroke patient who needs assistance during therapy, and it leads to the active-assisted case. The time-advanced mode models a patient with rapid hand movements; thus, the active-constrained case will be enabled. The performance of the proposed controller (OIC) is compared to the SOIC (Siciliano et al., 2009; Hogan, 2017) (see Appendix C), which is also designed for the robot to perform in four modes of the movement.

**Figure 3:**
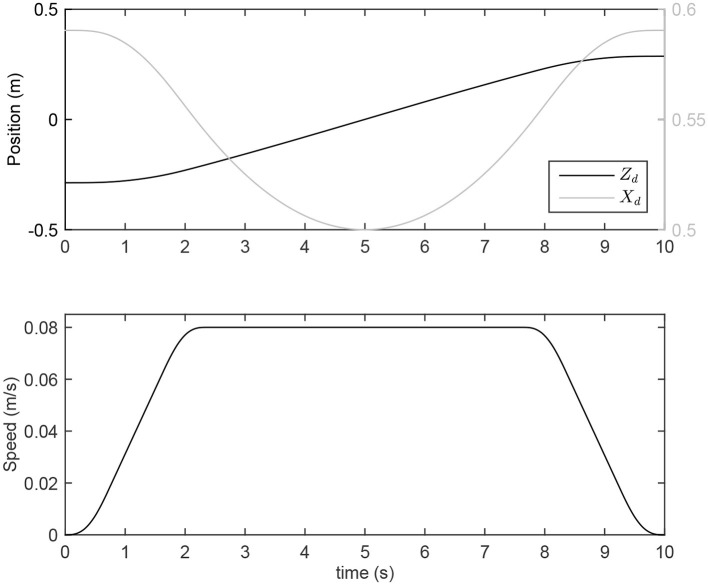
Desired position and speed for point-to-point reaching movement.

For LQR weights, matrix Q_Σ_ is defined to have less position error along the normal to the path, and matrix R_Σ_ is considered to have less force error along the path (see Appendix B for choosing LQR weights). To run simulations, we should consider a trajectory for manipulation. Based on Ghannadi et al. (2014b) the trajectory is approximated by a smooth curvilinear path with a large radius of curvature (Figure 1B). Then, a cubic spline interpolation approach is used to generate the path with a bell-shaped tangential speed profile and minimum jerk (see Figure 3).

Generated MapleSim™models are exported as optimized MATLAB® S-functions into the Simulink/MATLAB® environment. Sampling-time frequency of the simulations is set to ν = 1000 Hz, and a fixed-step Euler solver is selected to solve the ordinary differential equations.

### 3.2. Experiments

To evaluate the performance of the controllers during a rehabilitation procedure, a healthy male subject performed four modes of movement similar to the simulations (see Figure 1A). To this end, the following protocols are considered:

- Impaired-hand movement mode: the subject is asked to relax his/her upper extremity muscles and avoid any contractions as much as possible.
- Healthy hand movement mode: the subject should do his/her best in following the desired trajectory.
- Delayed hand movement mode: the subject is asked to follow a path that is delayed compared to the desired trajectory.
- Time-advanced hand movement mode: the subject should follow a path for which the desired trajectory is the delayed form.

In the last three protocols, the point on the curvilinear path at each simulation time step is defined by a small circular region. The subject should try to keep the end-effector position inside that region while tracing the curvilinear path. In other words, the small circular regions define the accuracy required for tracing the curvilinear path. To reduce the effect of random/noisy movements, each mode for each controller was performed in 10 trials. Tests of the two controllers were alternated randomly to reduce the effect of learning.

In our controller design, the optimality criterion is to minimize the tracking and applied force errors in the operational space. As discussed in the Introduction, the CNS optimally controls a human's environmental interaction. Hence, in a rehabilitation task, aligned with the CNS decision making process, the subject tries to minimize the interaction force and tracking error as much as possible. For the active-assisted (or active-constrained) therapy case, if the amount of assisting (or resisting) force can be increased with minimal change in the position tracking error induced by the subject's latency (or rapid movement), at first, the subject will decrease (or increase) muscle activations. However, later in the next stages of the therapy, unimproved tracking accuracy with higher interaction force will entice the subject to reduce the tracking error in order to decrease the applied interaction force, and this is done by increasing (or decreasing) muscle activations. In other words, in active-assisted therapy, an impaired subject may feel more assistive force if they are not able to minimize the tracking error. The increase in assistive (or resistive) force is regulated by the impedance model. In contrast to SOIC, this amount is achieved optimally in OIC based on the robot's configuration. That is why, later in the section 4, the optimal increase in interaction force in OIC is more significant than in SOIC.

The optimal increase in interaction force in OIC is in contrast to a conventional proportional-integral-derivative (PID) controller, which tries to minimize the tracking error while increasing the interaction force. With the PID controller, the subject will not have any motivation to minimize the tracking error, since the robot has already reduced it. Furthermore, if the subject suffers from stiff joints or muscle fatigue, the PID controller will increase his muscle activities, since he will resist against the robot movement and this may lead to injuries. It is worth noting that, in OIC, if the assistive (or resistive) force increases more than a certain amount so-called optimal interaction force, which is regulated by controller gains (similar to the PID controller), the tracking error will decrease and the subject will try to provide resisting force by increasing muscle activities.

Robot motors are rated at 115 mN-m of continuous torque. The continuous force at the hand (or end-effector) is limited to 13 N per plane of motion. Driven joint angles are measured by two optical encoders (with 4,000 count/revolution, which results in a sensitivity of 0.8 mm/count in detecting changes in the Cartesian space) connected to the motors. The disc and the belt mechanism increase the output torque by a ratio of 307/16. The robot end-effector has a 6-axis force-torque (FT) sensor, which measures the human-robot interaction forces and torques in body frame. The FT sensor has been calibrated to tolerate maximum 250 N on the horizontal plane and 1000 N normal to the plane. Sensor resolution is 1/24 N in the horizontal plane and 1/48 N normal to the plane.

The robot has frictional joints with stiffness and the manipulator moves on a frictional surface. These frictions are modeled using three continuous velocity-based frictional models (Brown and McPhee, 2016). Hence, robot dynamic Equation (1) is updated (refer to Ghannadi et al., 2017b for the detailed dynamic parameter identification of the robot):

(27) $$\begin{array}{c} T_{R}-J_{R}^{T}F_{int}=M_{R}(q)\ddot{q}+C_{R}(q,\dot{q})\dot{q}+K_{R}(q-q_{0})+f_{T}+J_{R}^{T}f_{F}=\;\Gamma_{R}(q,\dot{q},\ddot{q})\text{,} \end{array}$$

where **f**_*F*_ is the friction force under the end-effector in the global coordinates, **f**_*T*_ is the friction torque vector at the joints, and **K**_*R*_ is a 2 × 2 symmetric joint stiffness matrix.

The robot's computer software interface includes Simulink/MATLAB® which uses Quanser's real-time control software driver (QUARC). To control the robot, the driver software uses Quanser's data acquisition (DAQ) card (Q8). The driver and application software communicate through TCP/IP and shared memory protocol. To read the FT sensor data, a National Instruments DAQ card (PCI-6229) is used. This card is compatible and operable by the QUARC software. Sampling-time frequency of the experiments is set to ν = 500 Hz, and a fixed-step Euler solver is selected to solve the ordinary differential equations.

### 3.3. Assessment criteria

Muscle activities during reaching tasks in upper extremity rehabilitation have been used as a measure for performance evaluation (Wagner et al., 2007). As discussed, if the assistive (or resistive) force is greater than a threshold (i.e., optimal interaction force) then the subject will try to provide resisting force by increasing muscle activities, which may cause injuries. Maintaining this increase in the assistive (or resistive) force less than the threshold will decrease (or increase) muscle forces (i.e., activations); this is in good correlation with the goals of robotic rehabilitation therapy (Amirabdollahian, 2011). Therefore, to capture the effect of increased assistance (or resistance) to the subject, we assess the simulated performance of the OIC and SOIC controllers using the activation results from the musculoskeletal model interacting with the robot. Furthermore, the dynamic response of the system is used to evaluate the controllers in terms of the amount of interaction force and tracking error.

## 4. Results and discussions

### 4.1. Muscular activities

Since muscle activities less than 0.003 are mostly caused by suboptimal results and round-off calculation errors, muscles with activations less than this amount are not reported. Instead, active muscles with activations more than 0.003 are studied. These muscles are: Muscle 1, mono-articular shoulder flexor; Muscle 4, mono-articular shoulder extensor; and Muscle 5, bi-articular shoulder-elbow flexor.

**Figure 4:**
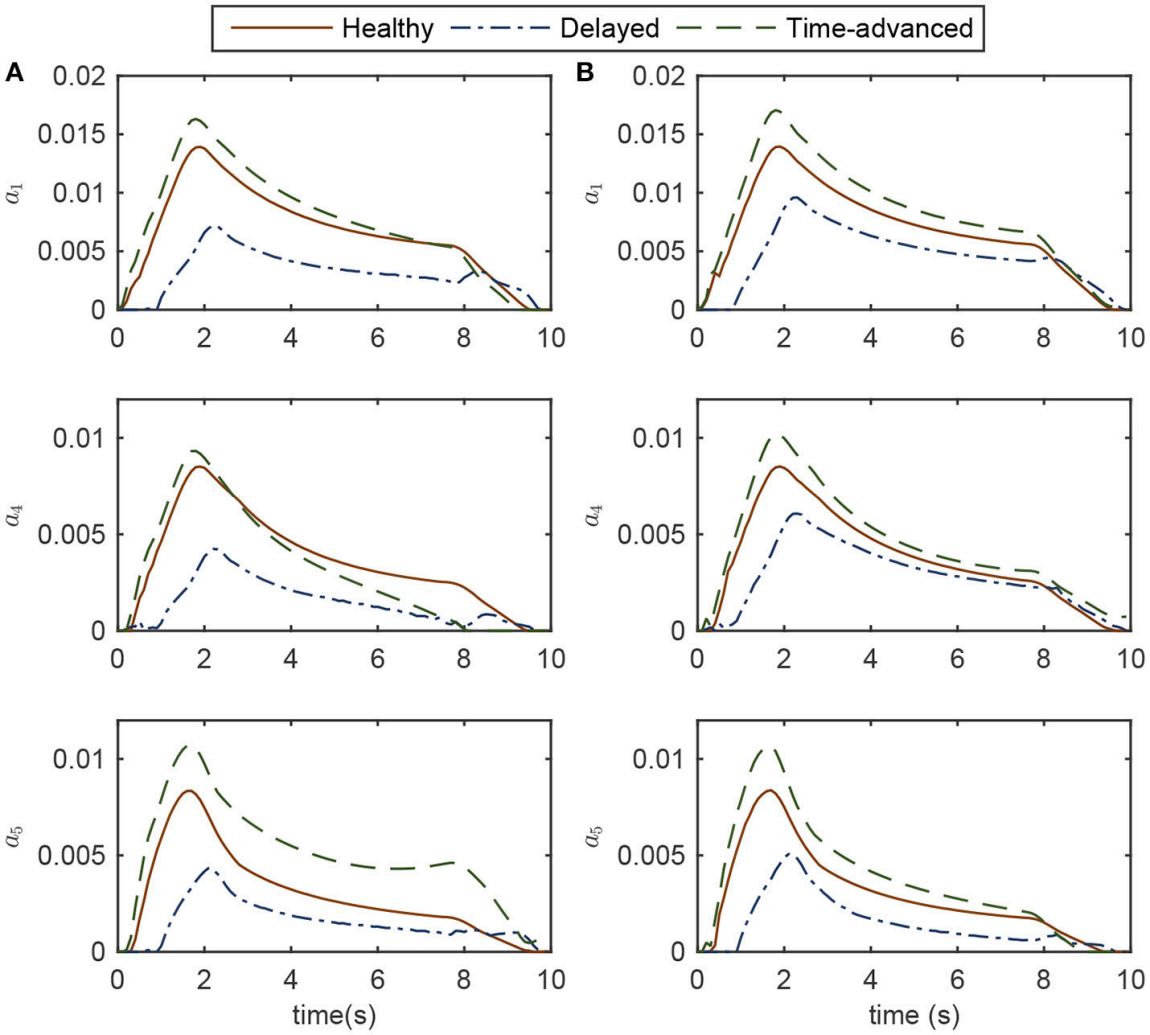
Activations of the active muscles in three modes of simulation while controlling the robot **(A)** OIC, and **(B)** SOIC. Note that in the impaired mode, muscle activations are zero.

As shown in Figure 4, for the delayed hand movement in both controllers, the robot assistance has decreased the amount of muscle activation compared to the other modes, for most of the path. In the delayed hand movement mode, when the robot detects a subject's latency, it increases the assistive force compared to the healthy hand movement. This increase in assistive force decreases the subject's muscular activities. Despite the increase in assistive force, the tracking accuracy has not improved. Thus, compared to the healthy subject, the subject with lower muscular activities will feel higher interaction force but unimproved tracking accuracy.

For the time-advanced mode in both robot controllers, the amount of maximum muscle activations is higher than the other modes. Similar to the delayed hand movement, despite the increase in resistive force, the tracking accuracy has not changed significantly. Therefore, compared to the healthy subject, the subject with higher muscular activities will feel more interaction force but with unimproved tracking accuracy. In the healthy hand movement mode, both controllers result in the same amount of activation.

Simulation results for both the delayed and time-advanced hand movements (in OIC and SOIC) indicate that active-assisted and active-constrained therapies have been invoked, respectively. In the active-assisted case, muscular activities of the subject are too low for the task to be finished independently; hence, the robot assists him. In the active-constrained case, the robot resists the subject with high muscular activities. From the delayed hand movement results (weak tracking performance with high interaction force), we can speculate that the subject will try to improve the tracking accuracy in the next stages of therapy by applying more muscle force, thereby decreasing the interaction force. Similarly, in the next stages of the therapy with time-advanced hand movement (more resisting force with increased muscular activities), the subject may try to reduce the tracking error and resisting force by decreasing muscular activities. In other words, continuing the robot therapy will achieve levels of muscle activation close to those for a healthy subject.

**Figure 5:**
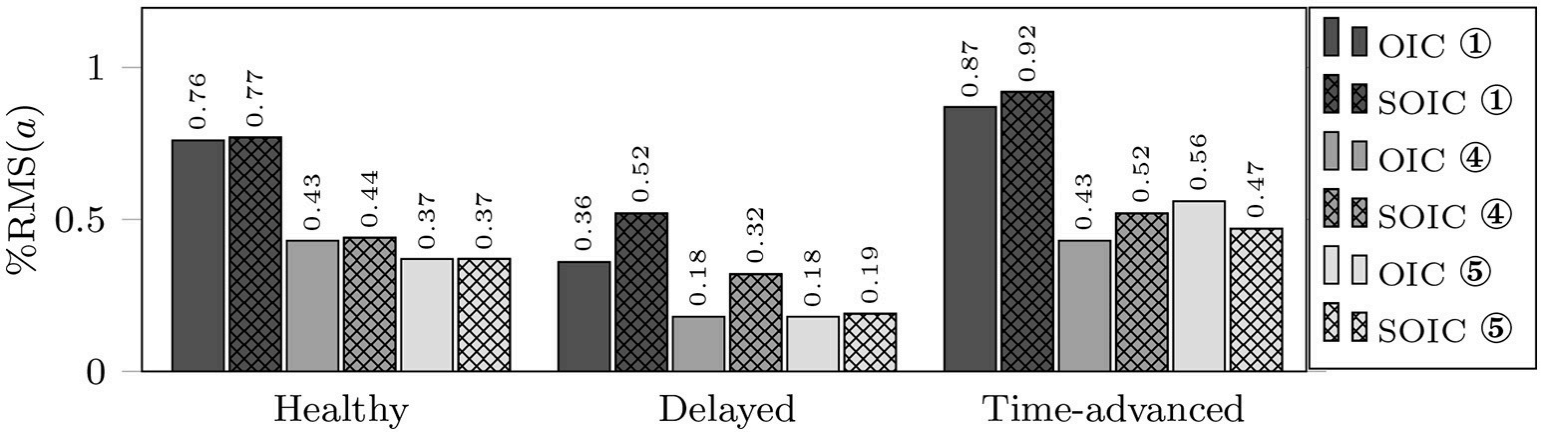
RMS of active muscle activations in three modes using OIC (solid fill) and SOIC (crosshatch fill). Circled numbers are corresponding to the active muscle numbers.

Although, both controllers are successful in implementing active-assisted and active-constrained therapies, in the delayed hand movement mode, the decrease in muscle activation for the OIC is more than the SOIC (see the root mean square (RMS) values for the delayed mode in Figure 5). Thus, in OIC, if the delayed hand movement subject wants to improve tracking accuracy and decrease the assistive force, he can have a wider range of muscle activations compared to SOIC. With a higher assistive force compared to the SOIC, and a wider range for the muscle activation changes, the OIC can be used for a wider range of applications and patients in active-assisted therapy. In other words, the OIC is more effective in active-assisted therapy.

### 4.2. Dynamic response

**Figure 6:**
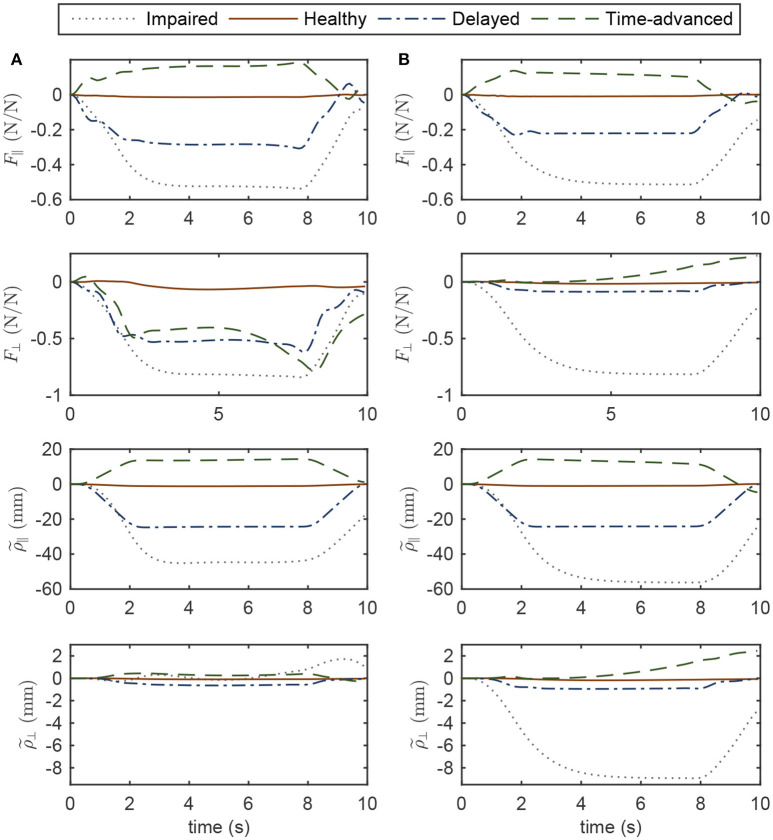
Operational space normalized interaction force and position error in four modes of simulations while controlling the robot with **(A)** OIC, **(B)** SOIC. Subscripts || and ⊥ indicate the tangent and normal directions, respectively.

**Figure 7:**
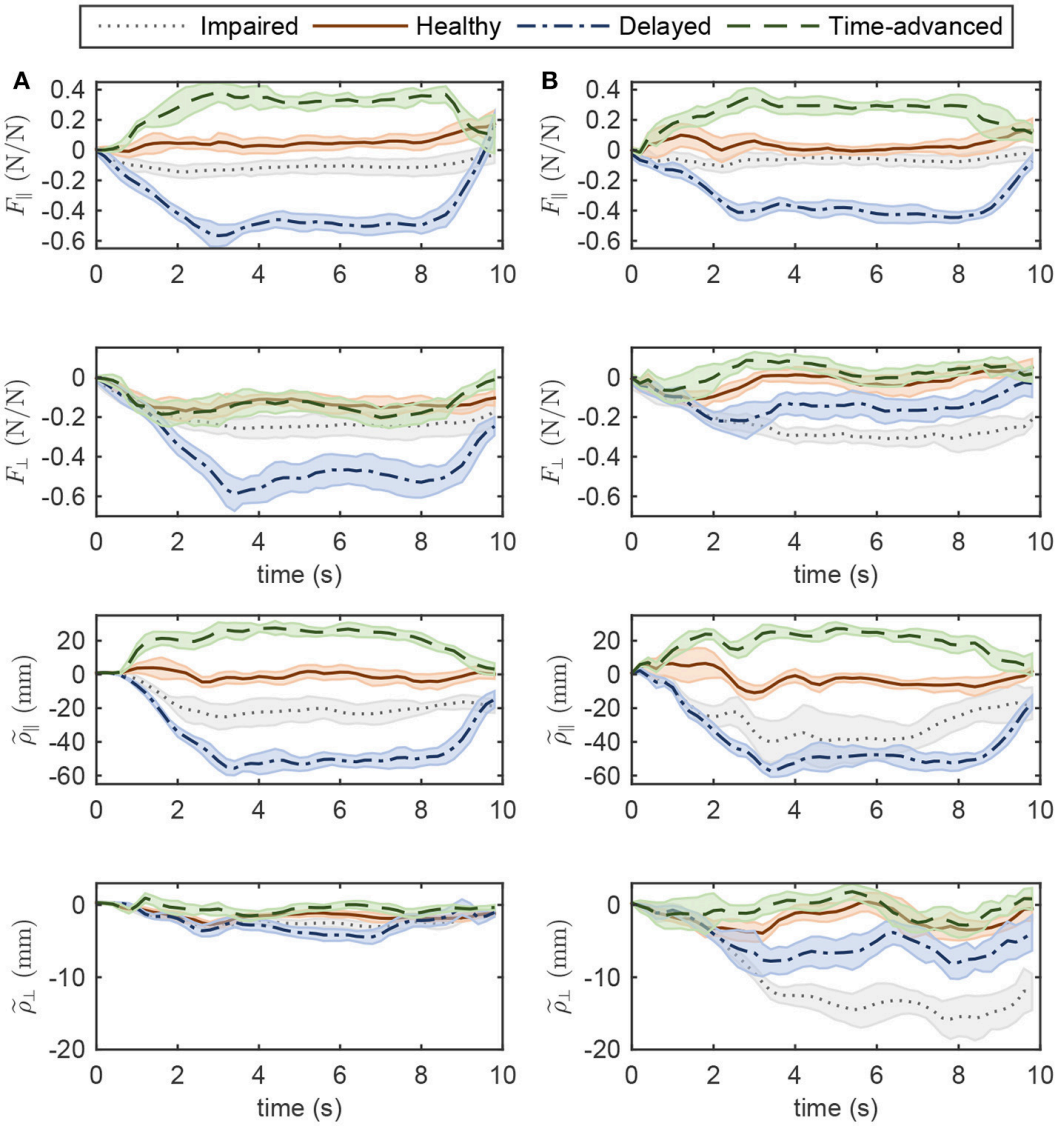
Operational space normalized interaction force and position error in four modes of experiments while controlling the robot with **(A)** OIC, **(B)** SOIC. The shaded area denotes twice the standard deviation at each instance of experiment.

Normalized interaction forces and position errors in the operational space are compared for four modes (see Figures 6, 7 for simulation and experimental results). Interaction force results are normalized to the maximum applied force in the horizontal plane to show similar trends to the approximate and highly idealized simulation model. Both in experiments and simulations, tangential interaction force plots show that the amount of assistance (in the delayed condition) or resistance (in the time-advanced condition) for the OIC is slightly more than the SOIC. Both in simulations and experiments, normal interaction force amount in the impaired hand movement mode for the OIC is not more than the SOIC, while for the other modes, the OIC results in higher values than the SOIC. This is because the position error in the normal direction is reduced by the OIC. However, the normal position error for the impaired hand movement mode in the SOIC is significantly more than the OIC. This shows that the optimal performance of the SOIC, especially in experiments, has failed to deal with impaired patients. The tangential position error is similar for both controllers.

In simulations with the SOIC (Figure 6), after 3 s of the simulation, normal position error for the time-advanced hand movement is strictly increasing, and this will result in instability issues. However, this does not happen in experiments, since robot instability limited the selection of higher gains for the SOIC. Thus, in experiments, the robot in the SOIC is set to be more compliant. In simulations for the SOIC, between the position error and the interaction force, there is a linear relationship which is due to the linear time invariant (LTI) impedance model of the controller. However, for the OIC this relationship is nonlinear, and this is because of the LTV impedance model of the controller. One cannot see this nonlinear relationship because the robot's frictional forces have changed the system behavior and made it linear.

Regarding the controller structure, for the OIC, the state-space model is controllable and observable because at each operational point (and any intermittent interval), C and O are rank 4; furthermore, the dominant pole position of the LQR controller (which is the closest eigenvalue of [*A*_**q**_ − *B*_**q**_*K*] to the imaginary axis) at each operational point has a negative real value, which makes the system critically damped. On the other hand, the optimum values of SOIC are such as to result in an under-damped system with a damping ratio of $\sqrt{2}/2$.

## 5. Conclusion

In this study, we designed and verified a modified LQR controller (i.e., OIC) for optimal impedance control, which indirectly considers the operational space and interaction forces. This modified LQR controller was compared to the SOIC (which is based on the feedback linearization approach). Despite some similarities to the SOIC, the OIC has proven to be more efficient in passive, active-assisted, active-constrained therapy since it updates the impedance gains optimally during a reaching task (at different robot configurations). Physiologically, this efficient behavior causes less muscle activations in active-assisted therapy. Dynamically, the controller is more robust to disturbances caused by unknown dynamics, and the tracking error and interaction force are in a safer region.

Since the QUARC software does not support online LQR gain adjustments using an LQR s-function during experiments, an offline gain selection is done based on the *desired* configuration of the robot. In online gain selection, the gains are updated based on the *current* configuration of the robot. Hence, in offline gain selection, the implemented controller can be classified as an optimal passive trajectory tracking controller. In recent experiments, we managed to perform online gain adjustment with MATLAB's built-in LQR controller, but the results were similar to the offline gain selection results presented in this paper. In the offline gain selection experiments, we generated different modes similar to the simulations. In other words, we maintained the current configuration of the robot close to the desired configuration. That is why, similar results from the offline and online gain selection experiments are obtained. The controller's computational cost is the same as that of the SOIC, even if the LQR gains are adjusted online. In OIC, therapists will be able to modify the controller with a single parameter *c* in (36), which represents the effort/state balance weight; the inclusion of a single calibration parameter contributes to the superiority of the OIC over SOIC.

Here, an integrated human-robot dynamic system is used to fine-tune the controller gains. This method is advantageous for efficient tuning of the robot controllers in experiments. A good qualitative agreement between experiments and simulations verifies the effectiveness of this method.

Our proposed controller and tuning method can be used in any rehabilitation manipulandum system. Possible improvements for this method are as follows. First, for a linear robot model, the OIC assumes an apparent mass for the robot equal to its mass matrix, while the SOIC permits offline changes to the robot's apparent mass. For considering the patient interaction dynamics, the robot's apparent mass should vary online as a function of the input frequencies of the system. However, neither the OIC nor SOIC offer such updates. Moreover, in regards to experiments, the unknown dynamics of the robot presents a challenging issue, independent of the controller. As a part of our future work, we will present a method to implement an OIC on the robot which also allows for online changes to the robot's apparent mass. Second, in the impedance model (8), the desired interaction force is assumed to be zero, while for implementing any high-level controller that deals with variable admittance environments (different patients or the same patients at different stages of their therapy) this desired interaction force should be updated by an outer-loop control law. In our future work, we will also develop the outer-loop controller to enhance the proposed OIC.

## Ethics statement

This study was carried out on a single subject in accordance with the recommendations of the Tri-Hospital Research Ethics Board (THREB) and the University of Waterloo Office of Research Ethics (ORE) with written informed consent from the subject. The subject gave written informed consent in accordance with the Declaration of Helsinki. The protocol was approved by the Tri-Hospital Research Ethics Board (THREB) and the University of Waterloo Ethics Board.

## Author contributions

BG development of the proposed method. RS technical help for conducting experimental trials. JM supervisor of the project.

### Conflict of interest statement

The authors declare that the research was conducted in the absence of any commercial or financial relationships that could be construed as a potential conflict of interest.

## Footnotes

^1^End-effector-based rehabilitation robots

^2^The compliance of tendon is proportional to its slack length. Thus, a tendon is compliant if its normalized slack length is large (≥ 10), and it is very stiff when it is equal to 1 (Zajac, 1989). For most muscles in the upper extremity this value is around 1; hence, the stiff tendon assumption seems to be valid.

^3^This research is focused on “Errand Completion Tasks” as opposed to “Time Management Tasks” (Sohlberg and Mateer, 2001), i.e., we assume that the timing in performing the rehabilitation task is not critical.

## Appendix A: satisfying the impedance model

By applying the control law (25) and (26) to the robot dynamics (27) and substituting **u**_*d*_ from (6), we get:

(A1) $$\begin{array}{c} \text{K}\;{\tilde{x}}_{q}+\;\Gamma_{R}(q_{d},{\dot{q}}_{d},{\overset{˙˙}{q}}_{d})-J_{Rd}^{T}F_{ext}=\;\Gamma_{R}(q,\overset{˙}{q},\overset{˙˙}{q})\text{.} \end{array}$$

Using Taylor series expansion and (13), (A1) can be rearranged as:

where:

The following equations can be derived from (20):

(A4) $$\begin{array}{l} {\tilde{x}}_{\text{q}}≊{\left[\begin{array}{c} \begin{array}{cc} J_{Rd}^{\Sigma } & 0\\ j_{Rd}^{\Sigma } & J_{Rd}^{\Sigma } \end{array} \end{array}\right]}^{-1}\\ {\tilde{x}}_{\Sigma }=\;\left[\begin{array}{cc} {\left(J_{Rd}^{\Sigma }\right)}^{-1} & 0\\ -{\left(J_{Rd}^{\Sigma }\right)}^{-1}j_{Rd}^{\Sigma }{\left(J_{Rd}^{\Sigma }\right)}^{-1} & {\left(J_{Rd}^{\Sigma }\right)}^{-1} \end{array}\right]{\tilde{x}}_{\Sigma }=T_{\Sigma }^{q}{\tilde{x}}_{\Sigma }, \end{array}$$

(A5) $$\begin{array}{c} {\tilde{\dot{x}}}_{\text{q}}≊{\dot{T}}_{\Sigma }^{q}{\tilde{x}}_{\Sigma }+T_{\Sigma }^{q}{\tilde{\dot{x}}}_{\Sigma }. \end{array}$$

Thus, (A2) can be written as:

(A6) $$\begin{array}{l} J_{Rd}^{T}F_{ext}=M_{Rd}\left[\begin{array}{c} 0\;I \end{array}\right]T_{\Sigma }^{q}{\tilde{\dot{x}}}_{\Sigma }\\ \;+M_{Rd}\left[\begin{array}{c} 0\;I \end{array}\right]{\dot{T}}_{\Sigma }^{q}{\tilde{x}}_{\Sigma }+\left[K_{{\;}_{P}}K_{D}\right]T_{\Sigma }^{q}{\tilde{x}}_{\Sigma }. \end{array}$$

(A6) is corresponding to the LTV impedance model (8), if:

(A7) $$\begin{array}{c} \{\begin{array}{c} M_{imp}={\left(J_{Rd}^{\Sigma }\right)}^{-T}M_{Rd}{\left(J_{Rd}^{\Sigma }\right)}^{-1},\\ B_{imp}={\left(J_{Rd}^{\Sigma }\right)}^{-T}K_{D}{\left(J_{Rd}^{\Sigma }\right)}^{-1}-2M_{imp}{\dot{J}}_{Rd}^{\Sigma }{\left(J_{Rd}^{\Sigma }\right)}^{-1},\\ K_{imp}={\left(J_{Rd}^{\Sigma }\right)}^{-T}K_{P}{\left(J_{Rd}^{\Sigma }\right)}^{-1}-\left(M_{imp}{\ddot{J}}_{Rd}^{\Sigma }+B_{imp}{\dot{J}}_{Rd}^{\Sigma }\right){\left(J_{Rd}^{\Sigma }\right)}^{-1}\text{,} \end{array} \end{array}$$

## Appendix B: choosing LQR gains

Matrices Q_Σ_ and R_Σ_ have diagonal weights:

(A8) $$\begin{array}{c} \{\begin{array}{l} \begin{array}{l} {\text{Q}}_{\Sigma }=\text{Diag}\left({\text{Q}}_{\Sigma 1},{\text{Q}}_{\Sigma 2},{\text{Q}}_{\Sigma 3},{\text{Q}}_{\Sigma 4}\right)\text{,}\\ {\text{R}}_{\Sigma }=\text{Diag}\left({\text{R}}_{\Sigma 1},{\text{R}}_{\Sigma 2}\right)\text{,} \end{array} \end{array} \end{array}$$

where these weights are chosen such that the cost function results in the allowable error associated with the state or effort, in other words:

(A9) $$\begin{array}{c} \{\begin{array}{l} \begin{array}{l} {\text{Q}}_{\Sigma ,i}=y_{tol,i}^{-2}\;(i=1..4)\text{,}\\ {\text{R}}_{\Sigma ,j}=cF_{tol,j}^{-2}\;(j=1..2)\text{,} \end{array} \end{array} \end{array}$$

in which *y*_*tol, k*_ and *F*_*tol, k*_ are the allowable amount of the *k*^th^ element of the state (${\tilde{\text{x}}}_{\Sigma }$) and effort (F_Σ_) vector errors, respectively. These weights should also be adjusted such that the (A7) results in positive definite impedance gains. Coefficient *c* > 0 will be controlled by the therapist to adjust the effort/state balance. The coefficients of the experiments for the OIC are given in Table 1.

**Table A1:** Experimental values of the OIC coefficients.

***y*_*tol*1_**	***y*_*tol*2_**	***y*_*tol*3_**	***y*_*tol*4_**	***F*_*tol*1_**	***F*_*tol*2_**	***c***
$\frac{1}{90}$ m	$\frac{1}{30}$ m	1 m/s	$\frac{100}{32}$ m/s	10 N	$\frac{10}{3}$ N	1

## Appendix C: standard optimal impedance control

For the robot dynamics (27), using nonlinear feedback linearization (inverse dynamics approach), we define the control law as (Siciliano et al., 2009):

(A10) $$\begin{array}{c} T_{R}=J_{R}^{T}F_{ext}+\;\Gamma_{R}(q,\dot{q},y), \end{array}$$

where y is the outer loop control law and is defined such that it changes manipulator behavior to a linear impedance under interaction force error. In other words, it is desired to have the linear impedance model in the operational space as in (8) with time invariant coefficients. This impedance model can be achieved if the outer loop control law is defined as:

(A11) $$\begin{array}{c} y=J_{R}^{-1}R_{\Sigma }M_{imp}^{-1}\left(M_{imp}(\dot{b}-{\dot{J}}_{R}^{\Sigma }\dot{q})+B_{imp}{\dot{\rho }}^{\Sigma }+K_{imp}\rho^{\Sigma }+F_{ext}^{\Sigma }\right)\text{,} \end{array}$$

where:

(A12) $$\begin{array}{c} \{\begin{array}{l} \dot{b}=R_{\Sigma }^{T}{\ddot{\rho }}_{d}-{\dot{\varpi }}_{\Sigma }{\tilde{\rho }}^{\Sigma }+\varpi_{\Sigma }\varpi_{\Sigma }{\tilde{\rho }}^{\Sigma }+\varpi_{\Sigma }R_{\Sigma }^{T}(J_{R}\dot{q}-2{\dot{\rho }}_{d})\text{,}\\ {\dot{J}}_{R}^{\Sigma }=R_{\Sigma }^{T}{\dot{J}}_{R}-\varpi_{\Sigma }R_{\Sigma }^{T}J_{R}\text{,}\\ {\dot{R}}_{\Sigma }=\varpi_{\Sigma }R_{\Sigma }\text{,}\\ \varpi_{\Sigma }=\left[\begin{array}{cc} 0 & -{\dot{\theta }}_{\Sigma }\\ {\dot{\theta }}_{\Sigma } & 0 \end{array}\right]\text{.} \end{array} \end{array}$$

For this controller, with a predefined diagonal mass coefficient matrix, the standard optimum stiffness and damping are as follows (Hogan, 2017):

(A13) $$\begin{array}{c} \{\begin{array}{l} M_{imp}=\text{Diag}\left(M_{imp1},M_{imp2}\right)\text{,}\\ B_{imp}=\text{Diag}\left(\frac{F_{tol1}}{y_{tol1}},\frac{F_{tol2}}{y_{tol2}}\right)\text{,}\\ K_{imp}=\text{Diag}\left(\sqrt{\frac{2F_{tol1}M_{imp1}}{y_{tol1}}},\sqrt{\frac{2F_{tol2}M_{imp2}}{y_{tol2}}}\right)\text{.} \end{array} \end{array}$$

The coefficients of the experiments for the SOIC are given in Table 2.

**Table A2:** Experimental values of the SOIC coefficients.

***M*_*imp*1_**	***M*_*imp*2_**	***y*_*tol*1_**	***y*_*tol*2_**	***F*_*tol*1_**	***F*_*tol*2_**
2.2 kg	2.2 kg	$\frac{1}{90}$ m	$\frac{1}{30}$ m	10 N	10 N
